# Surgical treatment of tailgut cysts: a single-center analysis of 19 cases

**DOI:** 10.1007/s00423-026-04348-0

**Published:** 2026-10-29

**Authors:** Petro Zgurskyi, Nina Schraps, Baris Mercanoglu, Ramez Wahib, Jakob R. Izbicki, Thilo Hackert, Nathaniel Melling

**Affiliations:** 1 Department of General, Visceral and Thoracic Surgery, University Medical Center Hamburg-Eppendorf, Martinistr. 52, 20246 Hamburg, Germany; 2 Department of Surgery, University Medical Center Schleswig-Holstein, Campus Lübeck, Lübeck, Germany

**Keywords:** Tailgut cyst, Retrorectal tumor, Rectal diseases, Hamartoma

## Abstract

**Purpose:**

Tailgut cysts are rare benign lesions with inflammatory and malignant potential. However, studies evaluating treatment outcomes in larger patient cohorts remain very limited. This study presents our institutional experience with tailgut cyst management, outlining diagnostic and therapeutic challenges and key insights gained.

**Methods:**

Retrospective, descriptive single-center study, including patients with tailgut cysts surgically treated from 2014 to 2025.

**Results:**

This study included 19 patients (female, 78.9%; median age, 35 years). The most frequent symptoms was lower back pain (36.8%). Meanwhile, 21% of the patients were asymptomatic. All patients underwent magnetic resonance imaging, with 78.9% suspected with a tailgut cyst or benign cystic lesion preoperatively. The mean cyst diameter was 5.8 ± 3.07 cm, and 36.8% of the patients had a multilobular morphology. Posterior (Kraske) approach was used in 78.9% of patients. One patient underwent laparoscopic cyst resection, and three required rectal resection. Posterior approach associated with minimal blood loss, lower complication rates and shorter length of stay. Regarding postoperative complication, wound infections was the most common (10.6%). Three (15,8%) patients experienced recurrence. The median follow-up period was 37 months.

**Conclusion:**

This study presents one of the largest single-center cohorts of resected tailgut cysts. Magnetic resonance imaging is typically sufficient for diagnosis. Multilobular cysts are associated with recurrence, often pose diagnostic challenges, and may lead to unnecessarily extensive surgical intervention, which is associated with an increased risk of complications. The dorsal approach combined with secondary wound healing proved safe and effective, yielding high patient satisfaction and low complication rates.

## Introduction

Tailgut cysts are rare benign lesions with a potential for superinfection or malignancy. They are often discovered incidentally and can pose significant challenges for both patients and healthcare providers. Among various presacral tumors, tailgut cysts are the most common [1]. However, these cysts are mostly reported in isolated case reports or small case series and rarely in large-scale studies offering cumulative clinical experience.

While some patients remain asymptomatic, others may present with wide-ranging clinical symptoms. Given the considerable heterogeneity of presacral tumors, including malignant lesions, as well as the variable location, size, and morphological heterogeneity of tailgut cysts, establishing an accurate diagnosis can be challenging [2]. Magnetic resonance imaging (MRI) is considered the gold standard and is often a reliable modality for differentiating between benign and malignant lesions [3]. Nevertheless, the role of biopsy remains controversial and is frequently debated both in the literature and in routine clinical practice [4]. Given the reported risk of malignant transformation of up to 8%, complete surgical resection remains the treatment of choice, including in asymptomatic patients [5, 6]. The selection of the surgical approach is largely determined by the size and anatomical location of the cyst [7]. The posterior approach is the most commonly employed; however, it has inherent limitations and has been associated with relatively high postoperative complication rates in some studies [8].

This study aimed to present our institutional experience in managing patients with tailgut cysts, highlight the challenges encountered throughout the diagnosis and treatment, and share the key insights gained.

## Materials and methods

This single-center retrospective study was conducted at the Department of General, Visceral and Thoracic Surgery, University Medical Center Hamburg-Eppendorf, Hamburg, Germany. The study conformed to the principles of the Declaration of Helsinki. Under German law, the use of data for research purposes is permitted without requiring written informed consent (HmbKHG, 12.1).

Between 2014 and 2025, 23 patients in whom a tailgut cyst was suspected and managed as such were identified. Following review, only patients with histologically confirmed tailgut cysts were included in the final analysis. Four patients were excluded: one with cystic teratoma, one with clear cell ovarian carcinoma, one with suspected tailgut cyst without intraoperative confirmation after prior surgery for a germ cell tumor, and one asymptomatic patient who declined surgical treatment. Nineteen patients with histologically confirmed tailgut cysts were included in the final analysis.

Patient identification and inclusion were conducted using both a prospective database and the histopathology registry to ensure comprehensive case capture. Data were extracted from electronic medical records via a standardized collection form by a surgeon, with independent verification of data accuracy performed by another surgeon. Patients’ medical records such as demographic data, clinical presentation, diagnostic findings, preoperative diagnosis, surgical approach, postoperative complications, and recurrence were reviewed.

Both anterior (transabdominal) and posterior (paracoccygeal) approaches were employed. The anterior approach was used in patients with suspected malignancy or when the cyst was located at the S1–S2 level. These patients underwent ultralow anterior resection with total mesorectal excision (TME) and creation of a protective stoma, or laparoscopic cyst resection, as appropriate. All procedures were performed in the lithotomy position.

**Fig. 1:**
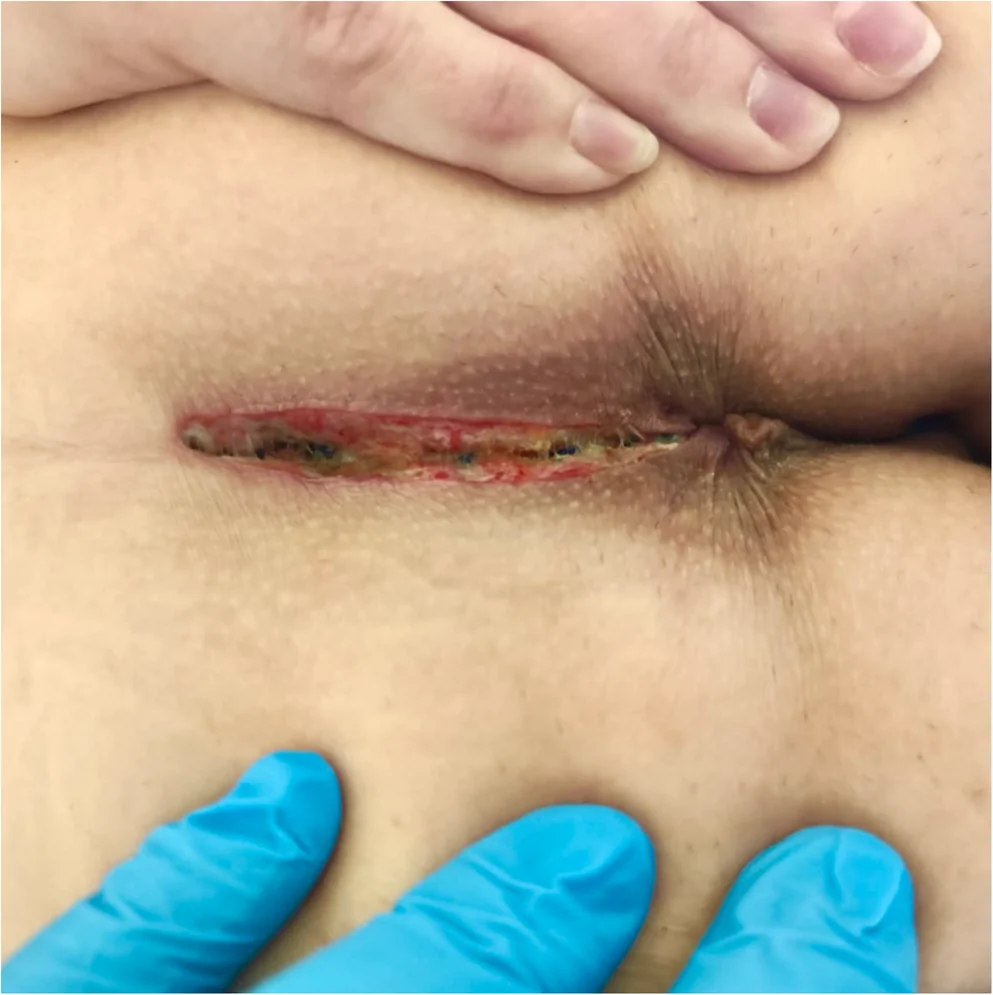
Postoperative wound on postoperative day 3

For the dorsal (posterior) approach, patients were positioned in the prone jackknife position. As originally described by Kraske in 1885, a midline incision with coccygeal bone removal is required to access the retrorectal space [9]. Coccygectomy was performed for large or more superiorly located cysts. Although this approach has undergone various modifications since its original description, the main steps have remained essentially unchanged [10]. After cyst mobilization and removal, a digital rectal examination was performed to ensure rectal integrity. The muscular layer was closed in a single layer using interrupted sutures to ensure adequate approximation of the wound edges. The subcutaneous tissue was similarly closed in a single layer using interrupted sutures. The skin edges were left open, with an approximately 5-mm gap, to allow for secondary intention healing. Patients were discharged home with instructions on wound care and hygiene. Figure 1 shows the postoperative wound on postoperative day 3, before the patient’s discharge. Wound infection was identified upon patient follow-up in the presence of pain, purulent exudate or wound discharge.

Follow-up was conducted via telephone interviews performed by one of the surgeons. Patients reporting symptoms were recalled for in-person evaluation, during which a clinical examination and further diagnostic work-up were performed. Recurrences of the disease were subsequently identified in these patients using MRI.

Owing to the small sample size, statistical analysis was not performed. Descriptive statistics are presented as mean ± standard deviation for normally distributed variables and as median with interquartile range (IQR) for non-normally distributed variables. Given the study’s descriptive nature, no statistical hypothesis testing was conducted.

## Results

This study included 19 patients. The majority were female (*n* = 15, 78.9%), with only four male patients (Table 1). The mean age during treatment was 35 years. Most patients were classified as ASA II (*n* = 15, 78.9%). Common comorbidities included thyroid disorders (21.1%) and endometriosis (10.5%). Ten (52.6%) patients previously underwent abdominal surgery. The median follow-up period was 37 (IQR, 5–60) months.

**Table 1:** Patient characteristics

Subcategory	Value
Total cohort, *n* (%)	19 (100%)
Male, *n* (%)	4 (21.1%)
Female, *n* (%)	15 (78.9%)
Age, median (IQR)	35 (24–58)
BMI, median (IQR)	24.2 (19.9–28.5)
ASA classification, *n* (%)	
● ASA 1	4 (21.1%)
● ASA 2	15 (78.9%)
● ASA 3	0 (0%)
● ASA 4	0 (0%)
Comorbidities, *n* (%)	
Hypo-/Hyperthyroidism	4 (21.1%)
Arterial hypertension	2 (10.5%)
Endometriosis	2 (10.5%)
Previous abdominal surgery	10 (52.6%)
Follow-up, in months, median (IQR)	37 (5–60)

*ASA* american society of anesthesiologists, *BMI* body mass index, *IQR* interquartile range

Most patients (78.9%) presented with symptoms, either as a single complaint or a combination of several. Lower back pain was the most frequent symptom, reported by approximately seven patients (36.8%). Lower abdominal or anal pain (at rest or during defecation) was observed in around one in five patients. Moreover, 15.7% of the patients had a history of chronic anal fistula. Hip and flank pain were rarely reported, occurring only in isolated cases (Table 2).

**Table 2:** Presenting symptoms

Subcategory	Value (%)
Total cohort, *n* (%)	19 (100)
Asymptomatic	4 (21.1)
Lower back pain	7 (36.8)
Lower abdominal pain	4 (21.1)
Perianal pain	4 (21.1)
Perianal fistula	3 (15.7)
Hip pain	2 (10.5)
Flank pain	1 (5.3)

When patients were referred to our tertiary institution for surgical treatment, most had already undergone extensive diagnostic imaging. All 19 patients received magnetic resonance imaging (MRI), with pelvic or abdominal imaging in 18 and hip imaging in one patient (Table 3). A preoperative diagnosis of a tailgut cyst or another benign cystic lesion was suggested in 15 (78.9%) patients. Furthermore, 4 (21.0%) patients underwent computed tomography (CT) of the abdomen. Image-guided biopsy was also performed in four patients. However, its diagnostic utility was limited, with epithelial cells identified in only one patient and the remaining samples regarded as nondiagnostic.

**Table 3:** Preoperative diagnostics

Subcategory	Value
Total cohort, *n* (%)	19 (100)
MRI (pelvis/abdomen)	18 (94.7)
● Suspected tailgut cyst	15 (78.9)
CT abdomen	4 (21.0)
Biopsy	4 (21.0)
Ultrasound abdomen	2 (10.5)
Colonoscopy	3 (15.8)
Endorectal ultrasound	2 (10.5)
MRI (hip)	1 (5.3)

*CT* computed tomography, *MRI* magnetic resonance imaging

**Fig. 2:**
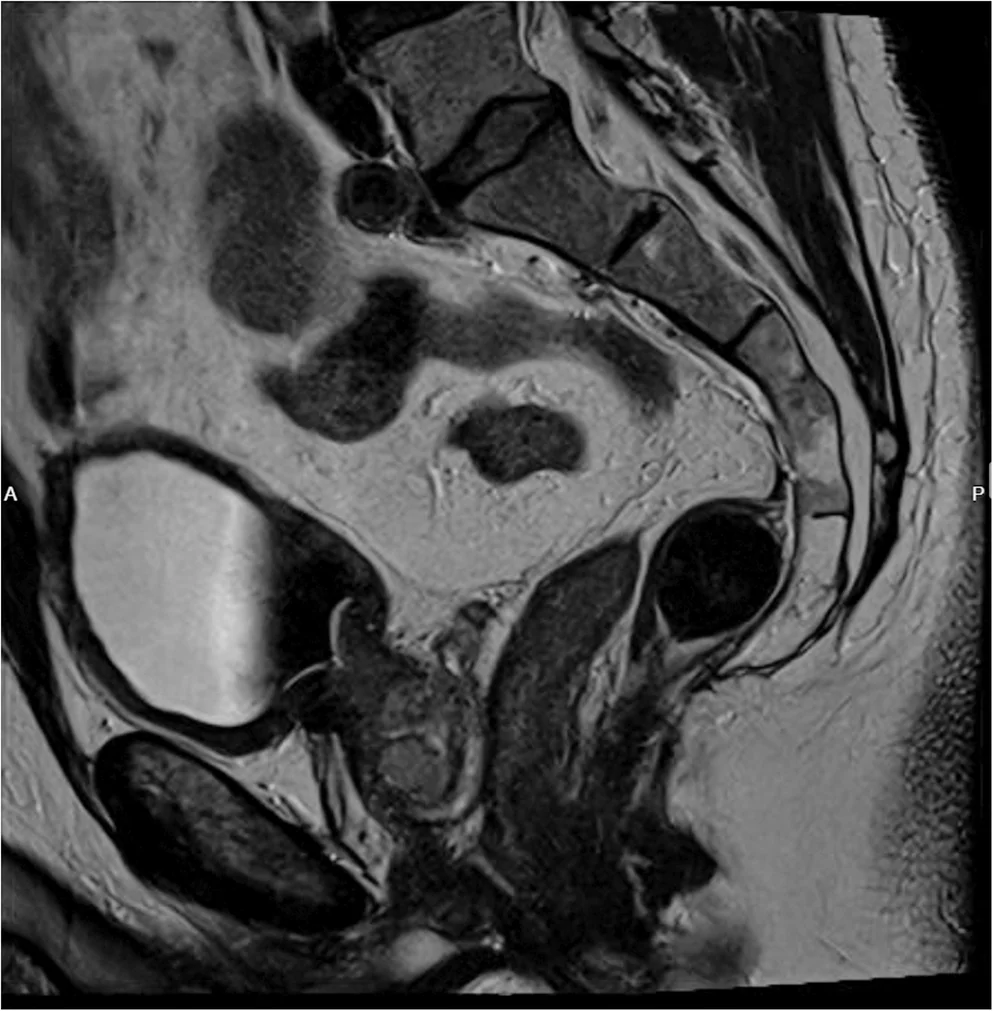
Cyst at the coccyx/S5 level

**Fig. 3:**
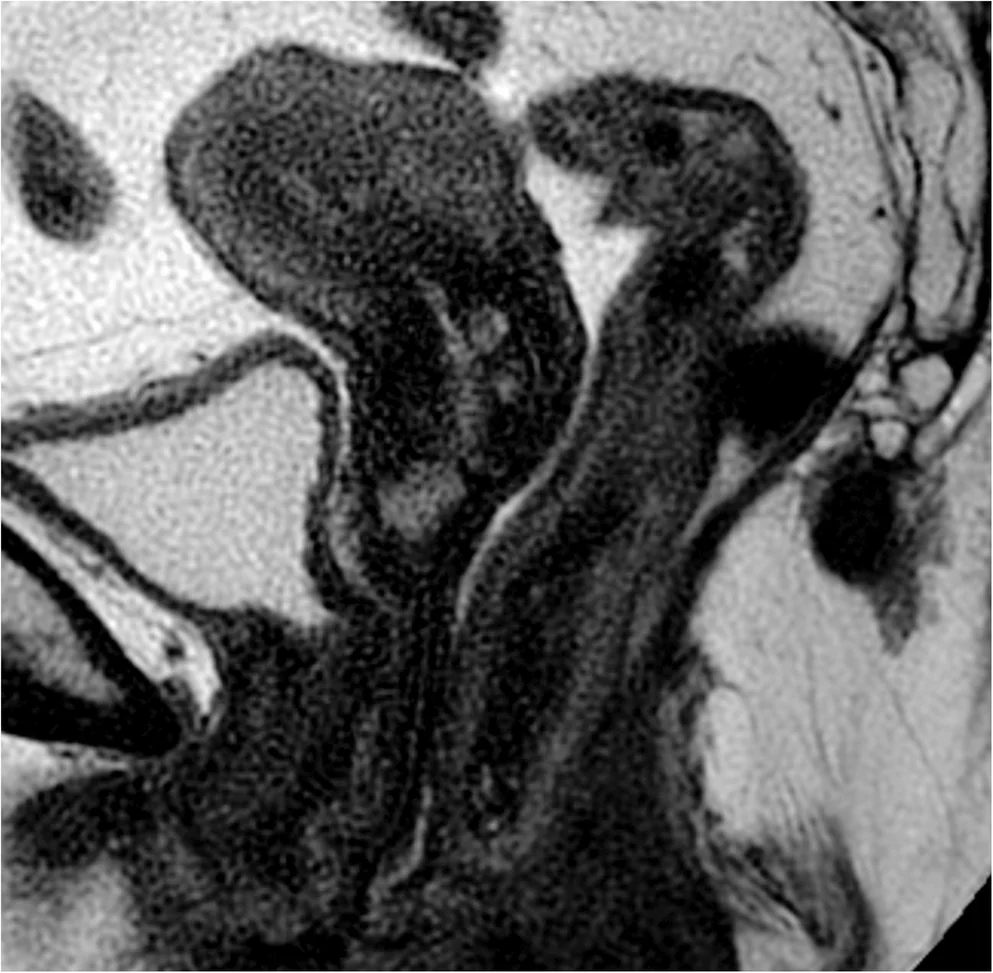
Two tailgut cysts

**Fig. 4:**
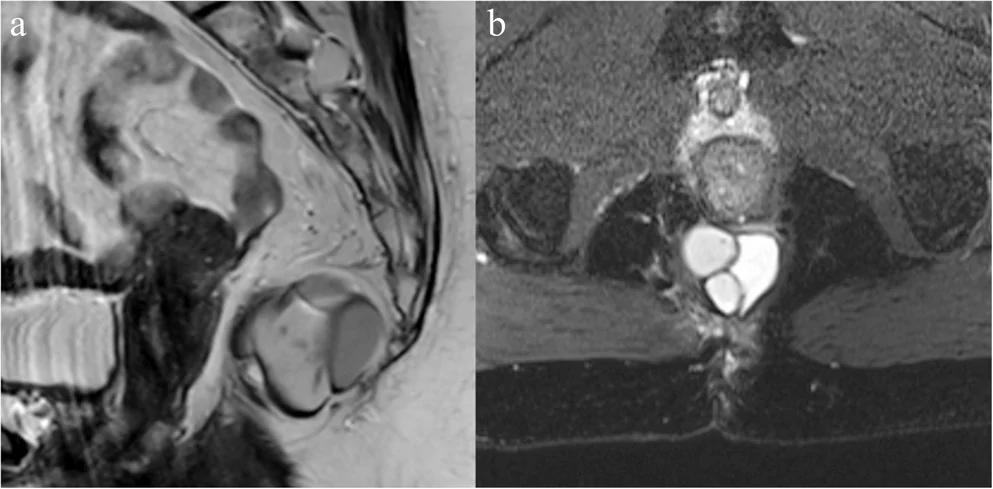
**a**, **b** Multilobular cysts

The mean cyst size was 5.2 cm, with the largest measuring 11 cm in diameter (Table 4). The cyst’s upper boundary most commonly corresponded to the level of the coccyx or S5 vertebra (Fig. 2). Two patients presented with multiple cysts (Fig. 3), and seven (36.8%) had multilobular cysts (Fig. 4a and b).

**Table 4:** Cyst characteristics

Cyst location, upper border	Value
● S2	1 (5.3)
● S3	5 (26.3)
● S4	1 (5.3)
● S5	7 (36.8)
● Coccyx	5 (26.3)
Multiple cysts	2 (10.5)
Multilobular cysts	7 (36.8)
Diameter, in cm, mean ± SD	5,2 ± 2,8

*SD* standard deviation

Meanwhile, 15 (78.9%) patients underwent resection using a posterior approach. More extensive procedures were required in four patients, as detailed below. All cases were discussed in a multidisciplinary team meeting.

A 22-year-old female patient presented with a cystic lesion in the lower pelvis that was misinterpreted for several years as a postoperative pseudocyst following neovagina formation for Mayer–Rokitansky–Küster–Hauser syndrome. Over a 3-year follow-up period, the lesion increased in size from 6 to 10 cm and became symptomatic. Given its high location at the S2 level and presumed benign nature, it was resected laparoscopically.

In a 49-year-old female patient, a multilobular cyst infiltrated the anal sphincter, raising a radiological suspicion of chordoma. Therefore, a radical oncologic resection was performed.

A 40-year-old pregnant patient at 16 weeks of gestation had a multilobular cyst with deep sphincter infiltration, raising concern for malignancy. Thus, an ultralow anterior resection with total mesorectal excision (TME) and diverting loop ileostomy was performed. Postoperatively, a spontaneous abortion occurred.

In a 74-year-old female patient, a large multilobular lesion containing solid components was observed, extensively invading the pelvic floor and pararectal fascia. She underwent ultralow anterior resection with TME. A deviating stoma was also formed. Postoperatively, she developed fecal incontinence, requiring permanent stoma (Clavien–Dindo grade 3b complication). After 8 years, she experienced a symptomatic recurrence and had developed a parastomal hernia. Surgical treatment included mesh hernia repair, rectal stump resection with en bloc resection of the recurrent tailgut cyst and coccyx via a combined anterior and posterior (Kraske) approach.

We would like to draw attention to one patient who was excluded from the study but was initially managed as a tailgut cyst. This 63-year-old female patient had a 9 cm lesion that could not be clearly differentiated between a tailgut cyst and an ovarian mass on MRI. Notably, she was the only patient to report night sweats. During dorsal resection, the lesion was found to be closely adherent to the lateral vaginal and rectal walls. Histopathological examination revealed clear cell ovarian carcinoma. Consequently, the patient underwent total hysterectomy, bilateral salpingo-oophorectomy, rectal resection, and adjuvant chemotherapy. No additional malignant findings were identified in the resected organs; however, the precise origin of the presacral ovarian tumor remains unclear.

In our cohort, 12 patients (63.1%) underwent coccygectomy to gain access to the pelvic floor and cyst (Table 5). However, in one patient, the cyst could not be identified intraoperatively. This patient had a chronic anal fistula and MRI had shown clear evidence of a cyst. Presumably, the cyst had drained into the fistula prior to surgery, preventing intraoperative detection. The residual cyst wall was removed, and the diagnosis was confirmed histologically.

**Table 5:** Comparison between the 2 surgical approaches and postoperative outcomes

Variable, *n* (%)	Anterior approach *n* = 4	Posterior approach *n* = 15
Size	4.1 (4–7)	3.7 (3–7)
Localisation		
● Above S3 (or opposite)	1 (25)	5 (33.3)
● Below S3	3 (75)	10 (66.7)
Multiple cysts	1 (25)	1 (6.7)
Multilobular cysts	3 (75)	3 (20)
Coccygectomy	0 (0)	12 (80)
No cyst found	0 (0)	1 (6.7)
Capsule violation	2 (50)	8 (53.3)
Organs involved		
● No organ involvement	0 (0)	6 (40)
● Rectum	4 (100)	9 (60)
Operative time, in min, median (IQR)	240 (240–292)	51 (37–62)
Blood loss, ml, mean ± SD	365 ± 475	0
Length of stay, days, median (IQR)	9 (7–14)	5 (4–6)
Wound healing complications	0 (0)	2 (13.3)
Clavien-Dindo Classification		
● 0	2 (50)	13 (86.7)
● I	0 (0)	1 (6.7)
● II	0 (0)	1 (6.7)
● IIIa	0 (0)	0 (0)
● IIIb	2 (50)	0 (0)
● IVa	0 (0)	0 (0)
● IVb	0 (0)	0 (0)
● V	0 (0)	0 (0)
Recurrence	1 (25)	2 (13.3)
Readmission	3 (75)	1 (6.7)

*IQR* interquartile range, *TME* total mesorectal excision

During surgery, cyst rupture occurred in 52.6% of patients. In 13 patients (68.4%), the cyst was densely adherent to the rectal wall, necessitating partial resection of the dorsal rectal muscular layer followed by repair. In the subgroup of patients operated via the posterior approach, the median operative time was significantly shorter at 51 min (IQR 37–62) compared to 240 min (IQR 47.7–172.2) for anterior approach. Procedures performed via the posterior approach were associated with minimal blood loss compared to larger resections. The median length of hospital stay after posterior resection was also shorter, at 5 days (IQR 4–6) versus 9 days (IQR 7–14). All 15 patients undergoing posterior resection received partial open-wound management to prevent superinfection. Postoperatively, only two patients (10.5%) in the posterior approach group developed septic wound complications, the most common complication in this study. One patient developed a purulent wound infection, requiring rehospitalization and intravenous antibiotics. In three patients who underwent anterior resection, closure of the protective stoma was performed after eight weeks. Cyst recurrence was observed in three patients (15,8%) at 2, 3, and 8 years postoperatively; all initially exhibited multilobular morphology. These patients presented with typical symptoms, confirmed by MRI, and were planned for reoperation, which was performed via the posterior approach again without further complications.

Out of 19 patients, intraoperative swabs were taken in 14 cases. Cultures were sterile in eight patients. In the six remaining patients, various enteric bacteria were identified, most commonly *Escherichia coli* and *Enterococcus faecalis* (Table 6).

**Table 6:** Postoperative microbiological findings

Subcategory	Value
Total cohort, *n* (%)	19 (100)
No swab taken	5 (26.3)
Sterile swab	8 (42.1)
Positive cultures included	6 (31.5)
● *Enterococcus faecalis*	2 (10.5)
● *Escherichia coli*	2 (10.5)
● *Enterobacter cloacae complex*	1 (5.2)
● *Coagulase-negative staphylococci*	1 (5.2)

## Discussion

This study represents one of the few large single-center series of tailgut cyst resections. Based on our experience, we propose the following conclusions: (1) MRI is usually sufficient for an accurate diagnosis and biopsy is not required. (2) The posterior (Kraske) approach, combined with secondary wound treatment is the most effective surgical technique in terms of success and complication rates for tailgut cyst resection. (3) Multilobular cysts are associated with greater diagnostic uncertainty, increased surgical complexity, and higher recurrence risk. (4) Intraoperative cyst rupture is common, but does not significantly contribute to postoperative infection or recurrence.

Our data show that tailgut cyst diagnosis is rather challenging. These cysts may be completely asymptomatic, while some patients report on various nonspecific complaints, or others present with chronic pararectal fistulas [11]. MRI is the most effective imaging tool for diagnosis of tailgut cysts, often rendering further diagnostic procedures or biopsies unnecessary. In a study reporting on results from the Mayo Clinic, radiologists could correctly identify the lesion as a tailgut cyst in 45% of cases [5]. In our center, the diagnostic accuracy was markedly higher (78.9%). Other larger studies have also discouraged biopsy of cystic lesions, reporting inaccurate results in up to 44% of cases [1]. In our experience, biopsy findings in cystic lesions were non-informative. Therefore, we do not recommend routine biopsy for cystic presacral lesions. When malignancy is suspected, the scenario becomes more contentious. In patients with solid components, CT-guided percutaneous biopsy is preferred because it can provide more specific information than MRI alone [4].

Consistent with previous reports, our findings confirm that multilobular morphology is common in tailgut cysts and may be linked to a higher recurrence risk [8, 12, 13]. In our cohort, all recurrences occurred in patients with initially multilobular cysts, possibly reflecting incomplete excision. A previous study presented a similar case wherein recurrence took place 2 years after the perineal excision of a multilobular cyst, without histological evidence of malignancy [14]. Furthermore, in our series, multilobular morphology frequently contributed to uncertainty in the preoperative diagnosis, which in some cases resulted in unnecessarily extensive surgical procedures, severe postoperative complications and further operations.

The choice of surgical approach depends on several factors, including the cranial extent and size of the cyst, the presence of infection, and the involvement of adjacent structures such as the rectum or vagina. To date, the most commonly reported technique is the posterior approach [2, 5], which aligns with our center’s experience. In most cases, complete resection of the tailgut cysts is possible using this approach. Coccygectomy may be required for adequate exposure, especially when the cyst’s cranial margin reaches the S3 level [5]. A Chinese expert consensus recommends the posterior approach for presacral cysts located below the S4 level [7]. Based on our experience, cysts located even at the S3 level can be successfully resected via a posterior approach. The advantages of this technique include low complication rates, insignificant blood loss, and short hospital stays. This approach is also suitable for patients who have previously undergone abdominal surgery, which accounted for 52.6% of cases in our series, as well as for patients with confirmed malignancy who may require major abdominal oncological resection in the future.

The most common postoperative complication in our cohort was wound infection. However, more severe complications, such as fecal incontinence requiring colostomy caused by sphincter injury, have also been reported [15]. Contrary to our results, wound infection rates reportedly reached as high as 60% following posterior approaches [8]. In our experience, leaving the surgical wound partially open postoperatively, as opposed to performing tight primary skin closure, is the key to minimizing infection risk because it allows for adequate drainage of the surgical site. While antibiotic therapy is rarely required, meticulous outpatient wound care and hygiene are essential. By establishing secondary wound healing, we achieved infection rates of 13.3%.

In our cohort, capsule rupture and spillage of cyst contents into the wound occurred frequently, but they did not correlate with increased wound infection rates, consistent with the data from the Mayo Clinic [5]. Intraoperative swabs often showed no pathogenic growth or revealed only intestinal commensal flora. Complete resection remains the most important therapeutic goal. To achieve optimal exposure, coccygectomy should be considered, as it may also contribute to a reduced risk of recurrence in cystic presacral lesions [16, 17]. However, this hypothesis could not be confirmed in our cohort.

Importantly, the posterior approach avoids injury to neighboring organs (e.g., rectum, vagina, and bladder) or facilitates their repair. Our data show that tailgut cysts frequently closely adhere to the rectal wall, likely resulting in wall injury. We recommend routine intraoperative proctoscopy, combined with an air-leak test and suture repair when needed. Inserting a white gauze into the rectum improves visual contrast, thereby assisting in early detecting injuries [8]. If suture repair of the rectum or vagina becomes necessary, the dorsal approach in the prone jackknife position offers optimal exposure and access to the involved organs.

### Limitations

This study has limitations, mainly the relatively small sample size and the short follow-up period for patients operated on shortly before publication. Given these constraints, statistical analysis between clinical variables cannot be performed. Nevertheless, this case series represents one of the few large single-center experiences with tailgut cysts reported to date.

## Conclusion

Tailgut cysts are rare congenital lesions that may present with various symptoms. MRI is typically sufficient for accurate diagnosis and does not require subsequent biopsy. Multilobular cysts are common and may complicate surgical decision-making, leading to more extensive procedures, and an increased risk of recurrence. Complete resection remains the most important goal. Performing the resection via the posterior (Kraske) approach allows for sufficient exposure of the retrorectal space, and the lesion can be completely excised. Additionally, open-wound management reduces the risk of postoperative complications, leading to shorter hospital stays.

## Data Availability

The raw data supporting the findings are available from the corresponding author upon reasonable request.
